# The fabrication of a novel polyacrylonitrile/reduced graphene oxide-amino-halloysite/bimetallic metal–organic framework electrospun nanofiber adsorbent for the ultrasonic-assisted thin-film microextraction of fatty acid methyl esters in dairy products with gas chromatography-flame ionization detection†

**DOI:** 10.1039/d0ra07674k

**Published:** 2021-04-21

**Authors:** R. Mirzajani, F. Kardani, Z. Ramezani

**Affiliations:** Chemistry Department, College of Science, Shahid Chamran University of Ahvaz Ahvaz Iran rmirzajani@scu.ac.ir +986113738044 +986113738044; Department of Medicinal Chemistry, School of Pharmacy, Ahvaz Jundishapur University of Medical Sciences Ahvaz Iran

## Abstract

In this work, electrospun polyacrylonitrile/reduced graphene oxide-amino-halloysite/bimetallic metal–organic framework (PAN/rGO-amino-HNT/Co_0.5_Zn_0.5_(MeIm)_2_) nanofiber film was synthesized and investigated as a novel adsorbent for the ultrasonic-assisted thin-film microextraction (UA-TFME) of fatty acid methyl esters (FAMEs), including palmitic methyl ester (PAME), oleic methyl ester (OAME), stearic methyl ester (SAME), and linoleic methyl ester (LAME), from dairy products. The hybrid nanocomposite was obtained *via* bonding halloysite nanotubes to reduced graphene oxide, followed by loading with bimetallic metal–organic frameworks. The determination of FAMEs with nanofiber film was performed in two stages of desorption and absorption where, initially, the analytes were adsorbed onto the nanofiber film and then desorbed with organic solvent. In this study, ultrasound was used for both the adsorption and desorption stages. The advantages of ultrasonication are extensive, overcoming the shortcomings of conventional techniques in terms of energy consumption and solvent use, allowing a shorter treatment time with a low cost of implementation. Based on PAN/rGO-amino-HNT/Co_0.5_Zn_0.5_(MeIm)_2_ thin film, a microextraction-gas chromatography-flame ionization detection (TFME-GC-FID) method was developed. Experimental parameters affecting the extraction and desorption steps were optimized. The desorption parameters, including desorption time and the properties of the desorption solvent, were investigated one factor at a time. Then, effective parameters in the adsorption step were optimized using a Box–Behnken design and Design-Expert 7 software. Under the optimal conditions, the method detection limits (S/N = 3) were in the range of 0.03–0.06 μg L^−1^ and the limits of quantification (S/N = 10) were within 0.11–0.23 μg L^−1^. The relative standard deviations for intra-day and inter-day precision were 2.4–4.7% and 2.6–3.4%, respectively. In the present work, the UA-TFME method was successfully applied for the quantification of fatty acid methyl esters in dairy products (milk, yogurt, cheese, yogurt soda and butter samples) for the first time. The fatty acids were transesterified using standard procedures and were subjected to UA-TFME treatment prior to GC-FID determination. The developed method possesses the advantages of simplicity, rapidity, cost-effectiveness, sensitivity, and non-invasiveness.

## Abstract 2

Electrospun polyacrylonitrile/reduced graphene oxide-amino-halloysite/bimetallic metal–organic framework nanofiber film was synthesized and successfully applied to the ultrasonic-assisted thin-film microextraction (UA-TFME) of fatty acid methyl esters from dairy products.

## Graphical abstract

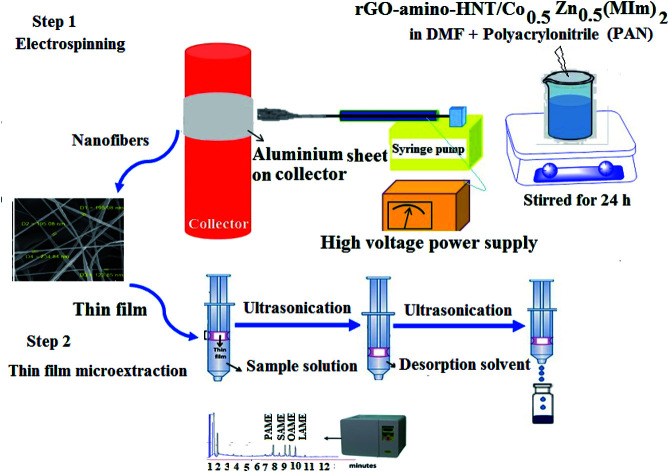

## 1. Introduction

Dairy products are milk and any of the foods made from milk, including butter, cheese, ice cream, yogurt, and condensed and dried milk. Milk has been used by humans since the beginning of recorded time to provide both fresh and storable nutritious foods. In some countries almost half the milk produced is consumed as fresh, pasteurized, whole, low-fat, or skim milk. However, most milk is produced into the more stable dairy products of global commerce, such as butter, cheese, dried milk, ice cream, and condensed milk. The premium nutritional quality of dairy products is highly correlated with milk fat quality and includes: a high concentration of fat-soluble vitamins and fatty acids, as well as a high content of conjugated linoleic acid (CLA). Quantification of free fatty acids in dairy products is not only important due to their (fatty acids') impact on the flavour and texture of dairy products but also because of their potential impact on nutrition and health, as well as for their anti-microbial properties. While milk and dairy foods are generally supposed to be healthy foods, milk fat comprises around 70% of saturated fatty acids (SFA). These fatty acids (mostly myristic and palmitic acids) may increase total and low-density lipoprotein (LDL) cholesterol, raising the possibility of cardiovascular disease (CVD), and thus the consumption of milk fat should be limited.^1–3^

Fatty acids can be categorized into short chain,^2–8^ medium chain,^8–12^ and long chain^13–24^ or into saturated, monounsaturated, and polyunsaturated fatty acids (SFAs, MUFAs, LCFAs and PUFAs, respectively) based on the existence or absence of double bonds. PUFAs such as linoleic acid (*cis*-9,12-octadecadienoic acid, C_18_) and α-linolenic acid (*cis*-9,12,15-octadecatrienoic, C_18_) are ‘vital fatty acids’, as the human body is not able to synthesize them, and they are only provided *via* the diet. Palmitic and stearic acids are other important PUFAs which exist in the lipids of most organisms.^4–7^ Thus, an essential step in the detection and control of the fatty acids of dairy products in human life is to employ a simple, accurate, and practicable method. The most commonly used method for analyzing FAs involves determining relevant fatty acid methyl esters (FAMEs) using capillary gas chromatography (GC) with a flame ionization detector (FID).^8,9^ For determining FAs in lipids, fats, and oils, a transesterification procedure is often performed, involving direct conversion of FAs to alkyl esters (particularly methyl esters) by alcohol in the presence of a catalyst. The derivatization procedure (especially for the longer chain FAs) is mandatory for enhancing the volatility and overcoming the adsorption of the polar functional groups onto the GC column.^10,11^ Thermally labile FAs can also be separated using high performance liquid chromatography (HPLC), capillary electrophoresis, and supercritical fluid chromatography.^12^

Nevertheless, sample preparation methods are often required prior to analysis by these instruments. Recent tendencies in analytical chemistry include simplification, automation, and miniaturization of the sample pretreatment, as well as the reduction of organic solvents consumed.

Solid-phase microextraction (SPME), originally introduced by Pawliszyn in the early 1990s, has become one of the main widely used microextraction methods due to its excellent advantages, including simple operation and field usability, and being time-saving as well as solvent-free.^13^ The common format of SPME is a rod fiber geometry but sometimes the usage of SPME becomes limited. As a result, low amounts of analytes are extracted by the rod fiber, particularly in the case of a complex matrix, which may cause relatively low sensitivity. Enhanced extraction capacity can be obtained by increasing the thickness of the fiber coating, though it will lead to the prolongation of the extraction equilibrium. In order to improve the sensitivity and extraction capacity, a new format of SPME, thin film microextraction (TFME), has been introduced. In this method, flake-like films with a large surface area-to-volume ratio are used as extraction phases to extract analytes. Thus, enhanced extraction capacity and a reduced time of extraction equilibrium are attained for the technique. Because of its beneficial properties, this technique has been utilized for determining diverse compounds. Thin films are usually prepared using an electrospinning technique. They typically have dimensions ranging from submicrons to nanometers. In analytical chemistry studies, these thin films have been used as an extraction phase for the extraction of a wide range of compounds using various methods such as solid phase extraction (SPE),^14^ SPME^15^ and semi-micro-SPE.^16^

Halloysite nanotubes (HNT) are a natural mineral nanosized tubule with the general formula AlSi_2_O_5_(HO)_4_·*x*H_2_O. This compound contains 15 to 10 layers of alumina silica tubes. The presence of a layer of water molecules at intervals between each layer creates a gap of 0.1 nm. The halloysite is composed of octahedral layers of octo-3 Al(OH) and tetrahedral SiO_4_ tubular layers. Various layers are bonded *via* hydrogen bonding between the oxygen atom of the tetrahedral layers and the OH group in the octahedral layers. The halloysite has important potential applications in various industrial and research fields. Indeed, this compound can be used in pharmaceutical directories and catalysts. The halloysite has a one-dimensional porous cellular structure at meso-pressure scale, 2–50 nm, which is larger than the porosity of most synthetic porous materials including carbon nanotubes. It also has high compatibility and low toxicity, and can be utilized for cases such as encapsulating biological molecules. The thermal stability above the halloysite tube structures should be significant for the design of a halloysite or ceramic base nanocomposite.^17,18^ Metal–organic frameworks (MOFs) are an interesting group of zeolite-like compounds synthesized by metal ions or clusters chelated with organic ligands. According to the accessibility of diverse metal ions, organic ligands, and the chelating ratio difference, MOF structures have demonstrated excellent properties, such as tunable pore sizes and large surface areas.^19,20^ Their unique characteristics make MOFs promising for different applications in analytical chemistry. MOFs have also been shown to be effective sorbents for sampling, SPE, SPME, and as stationary phases for gas and liquid chromatography. Heterometallic MOFs have been investigated for modifying framework properties such as enhancing framework stability, gas sorption activity, and catalytic behavior, as well as tuning breathing activity, luminescence, and magnetic features.^21^ A general strategy toward heterometallic MOFs, especially bimetallic MOFs, is to use two different metal ions as reactants during the conventional solvothermal reaction process. The reaction of a ligand with two different metal ions sometimes results in a bimetallic MOF as a pure phase rather than a mixture of two homometallic MOFs.^22,23^ Nevertheless, most MOFs have some drawbacks, including poor chemical stability, which must be overcome if they are going to find practical utility under industrial conditions.^24^ Recently, a considerable body of research has suggested that combining MOFs with different functional materials, such as carbon nanotubes (CNTs), graphene oxide, graphite oxide, and carbon nanofibers to prepare different composites, can significantly enhance their performance.^25–27^ GO is composed of distorted graphene layers, carbonyl, hydroxyl, and epoxy groups on the basal planes and carboxylic functionality on the edges of the carbon sheets.^28^ All of these mentioned factors acidify the material and make it highly hydrophilic, which explains its easy delamination in alkaline or alcohol solutions.^29^ Owing to its chemical structure, GO has been utilized in the preparation of various kinds of composite materials with better electronic and adsorptive properties.^30^ In this study, reduced graphene oxide (rGO) was prepared and used as substrate. rGO is obtained through the chemical reduction of GO. Indeed, rGO, a monolayer of sp^2^ hybridized carbon atoms arranged in hexagonal rings, has attracted extensive research interest due to its unique properties over the last decade. The hydrophobic surface of rGO makes it suitable for separating organic substances through π interaction. Meanwhile, the hydrophobicity and strong van der Waals interactions of rGO lead to insolubility and difficult dispersion in aqueous solutions.^31^ Thus, several inorganic nanoparticles (NPs) containing clay particles have been attached to GO and the rGO basal plane to modify the surface features.^32,33^ The present work, for the first time, deals with the synthesis of a newly designed electrospinning thin film nanocomposite with HNT, rGO, and bimetallic-MOF for ultrasonic-assisted thin film microextraction (UA-TFME). Then, to show the extraction efficiency and development of the analytical potential of GC-FID, it was used for the quick extraction of four banned dietary fatty acids of dairy product (milk, yogurt, cheese, yogurt soda and butter) samples. The proposed method has advantages including rapidity, simplicity, sensitivity, good extraction efficiency and low-cost sample preparation with an acceptable efficiency, as well as a low matrix effect compared with other techniques reported for determining fatty acid methyl esters.

## 2. Experimental

### 2.1. Reagents and materials

(C_14:0_, 99–100%), palmitic (C_16:0_, 99%), oleic (C_16:1_, 99%), stearic (C_18:0_, ≥99%), and linoleic (C_18:2_, 99%) acids and polyacrylonitrile (PAN), were purchased from Sigma-Aldrich (https://www.sigmaaldrich.com, USA). Methanol (HPLC grade), boron trifluoride and *n*-hexane were purchased from Merck (Darmstadt, Germany, http://www.merck.com). Zn(NO_3_)_2_·6H_2_O, Co(NO_3_)_2_·6H_2_O, 2-methylimidazole (MeIm) and *N*,*N*-dimethylformamide (99.5%, DMF) were provided by Sigma-Aldrich and used for bimetallic ZIFs (Co_*x*_Zn_1−*x*_(MeIm)_2_) synthesis. Halloysite nanotubes (1–3 μm length, 30–70 nm inner diameter, 64 m^2^ g^−1^ surface area) were purchased from Sigma-Aldrich. Aminopropyl triethoxysilane (APTES), graphite, sodium hydroxide, hydrochloric acid, hydrogen peroxide 30% and sodium chloride were purchased from Merck. Toluene, cyclohexane, *n*-hexane, dichloromethane, chloroform and all other chemicals were obtained from Merck. The real samples used for this study were filtered through a nylon 0.45 μm filter before use. Dairy samples were purchased from local supermarkets then stored at 4 °C prior to use.

### 2.2. Apparatus and instruments

The analysis was performed with a gas chromatograph (model GC-2550TG, Teif Gostar Fraz Co. Ltd, Iran) equipped with a flame-ionization detector (GC-FID) and a BP5 capillary column (25 m × 0.32 mm I. D. film thickness 0.5 μm). The column temperature was programmed as follows: initial oven temperature 40 °C for 5 min, increasing to 100 °C at 10 °C min^−1^ and directly to 200 °C at 20 °C min^−1^ then holding for 1 min. The injection port temperature was set at 250 °C and analysis of analytes was carried out in the splitless mode for 20 s, plus 5 additional minutes with the split valve on, for complete removal of analytes. The FID temperature was held at 260 °C. High-purity hydrogen (99.999%) was used as the carrier gas with a flow rate of 1 mL min^−1^. Additional make-up gas was also high-purity nitrogen with a flow rate of 60 mL min^−1^.

Scanning electron microscopy (SEM) analysis was performed with a Hitachi S5200 Instrument (Tokyo, Japan) in scanning mode at 30 kV. The chemical compositions were determined by an energy dispersive X-ray spectrometer (EDX) attached to the SEM instrument. The infrared absorption spectrum to identify the functional groups and chemical bonding of the rGO-amino-HNT/Co_0.5_ZnO_0.5_(MeIm)_2_ was obtained on a KBr tablet using a BOMEM MB-Series 1998 FT-IR spectrometer. XRD were recorded using a Bruker D8 Advance X-ray powder diffractometer with a CuKα radiation source (*λ* = 0.154056 nm) generated at 40 kV and 35 mA. An ultrasound water bath (Bandelin Sonorex, RK103H, 140/560 W, 35 kHz, Germany) was used for both adsorption and desorption stages. PTFE filters (SCA grade, 0.45 μm, 25 mm, CHMLAB, Barcelona, Spain) were applied for the filtration of the aqueous and organic solvents prior to use.

### 2.3. Sample preparation

The real samples used included the dairy products: milk, yogurt, cheese, yogurt soda and butter. To prepare the milk and dough samples, 1 mL of 3% acetic acid was added to 10 mL of milk and kept in a refrigerator at 4 °C for 30 minutes. Then it was centrifuged for 15 minutes and the solution was filtered. A subfiltration solution was used for the transesterification procedure. To prepare yogurt and cheese, almost 1 g of the sample was weighed and poured into a centrifuge tube and then 15 mL of trichloroacetic acid 1% and 5 mL of acetonitrile were added. After sonication for 30 minutes, it was placed on a vertical shaker for 10 min and centrifuged for 10 min at 4000 rpm. A filter paper was wetted with 5% trichloroacetic acid in water, and the supernatant was filtered using filter paper and distilled into 10 mL volume. This mixture was vortexed and finally 5 mL of the subfiltration solution was used for the transesterification process. No specific preparation process was performed on the butter. The transesterification process was carried out by mixing oil (15 mg) with 0.5 M sodium methanoate (1.25 mL) in a vial (16 mL). The vial was tightly capped and was heated for 5 min at 60 °C. The vial was then cooled and 14% boron trifluoride in methanol (2 mL) was added, and heated (80 °C) with continuous stirring for 60 min. The vial was allowed to cool and the mixture was then diluted to the 100 mL mark with methanol (1000 μg L^−1^). The solution was used for different extraction steps.

### 2.4. Synthesis of ZIFs (Co_*x*_Zn_1−*x*_(MeIm)_2_)

**Fig. 1:**
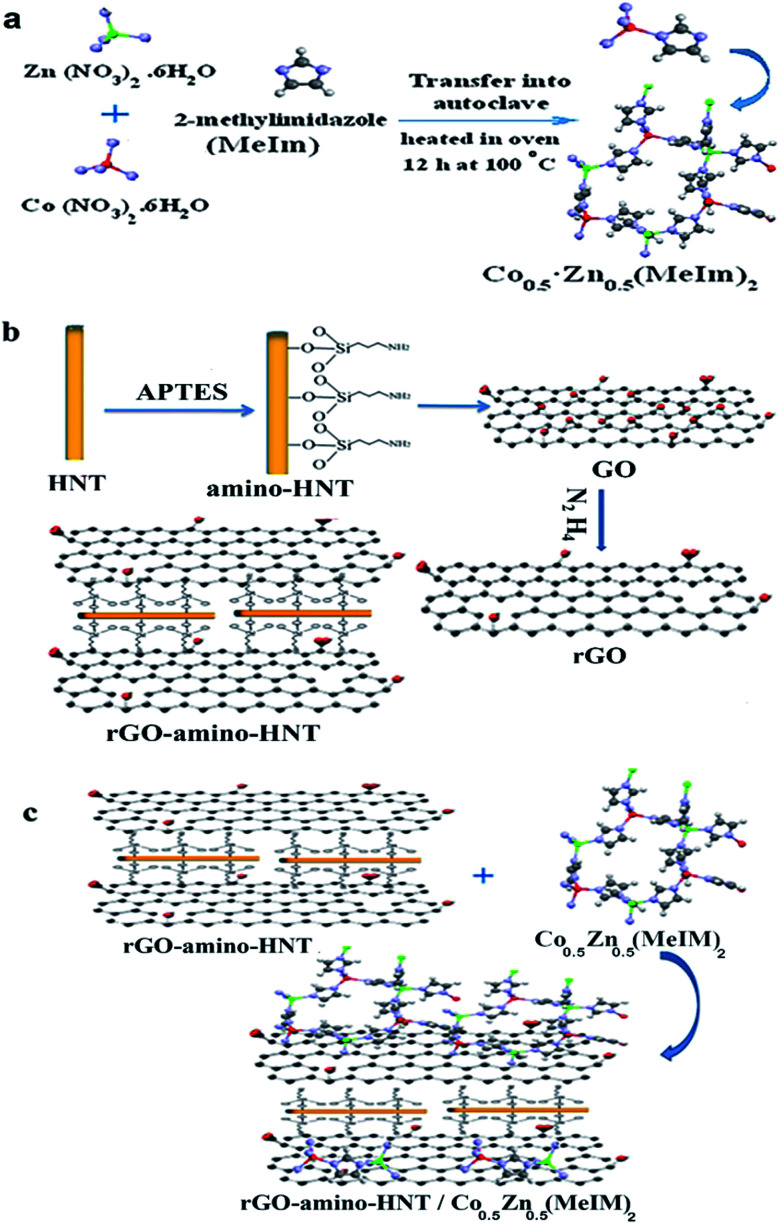
The overall strategy used for the preparation of the rGO-amino-HNT/Co_0.5_Zn_0.5_(MeIm)_2_ nanocomposite.

For the synthesis of bimetallic ZIFs (Co_*x*_Zn_1−*x*_(MeIm)_2_), Co(NO_3_)_2_·6H_2_O and Zn(NO_3_)_2_·6H_2_O (1.5 g) were dissolved in 30 mL of methanol while being shaken, after which a certain amount of 2-methylimidazole (3 g) was dissolved in 10 mL of methanol and then added to the above solution, and sonicated once for 10 min. The solution was transferred to a 100 mL stainless steel autoclave and placed in an oven for 12 h at 100 °C. Once the autoclave had cooled, the crystals were collected through centrifugation and washed with methanol and then dried at 60 °C. To prepare the bimetallic ZIF (Co_*x*_Zn_1−*x*_(MeIm)_2_) MOF, different ratios of two metals including Co_0.05_Zn_0.95_(MeIm)_2_, Co_0.1_Zn_0.9_(MeIm)_2_, Zn_0.67_Co_0.3_(MeIm)_2_, Co_0.67_Zn_0.33_(MeIm)_2_ and Co_0.5_Zn_0.5_(MeIm)_2_ were used. The obtained microscopic images clearly showed that the ratio Co_0.5_Zn_0.5_(MeIm)_2_ had a regular geometric hexagonal shape while the other ratios did not reveal a regular geometric shape. Thus, this ratio was used for subsequent stages of thin film preparation.^22^ The strategy applied for preparing the preparation of the bimetallic ZIF (Co_*x*_Zn_1−*x*_(MeIm)_2_) metal–organic framework is shown in Fig. 1.

### 2.5. Synthesis of rGO-amino-HNT

Initially, GO was synthesized according to the method described in ref. 31. Then an appropriate amount of synthesized GO was dissolved in 10 mL of ethanol–water (1 : 1 v/v), and then dispersed for 30 min in an ultrasonic bath. Next, rGO-amino-HNT was prepared by adding 1.5 g of amino-HNT (synthesized according to the method in ref. 34) to 50 mL of water/ethanol (DMF/EtOH) solution (40 mL, v/v, 1 : 1), and then dispersed in an ultrasonic bath. Then, the GO dispersion solutions were added sequentially to amino-HNT solution and stirred at 60 °C for 1 h. Subsequently, the temperature was elevated to 90 °C, after which 0.3 mL 85% N_2_H_4_ was added to the mixture, along with stirring for 30 min. The solid product was collected, washed thoroughly with water/ethanol (DMF/EtOH), and finally dried at 50 °C for 2 h in an oven (Fig. 1).

### 2.6. Synthesis of rGO-amino-HNT/Co_0.5_Zn_0.5_(MeIm)_2_

The bimetallic rGO-amino-HNT/Co_0.5_ZnO_0.5_(MeIm)_2_ MOF was synthesized by the hydrothermal method. Firstly, 0.5 g of Co(NO_3_)_2_·6H_2_O and Zn(NO_3_)_2_·6H_2_O were each added to 30 mL of methanol. Then the mixture was sonicated for 10 minutes until a smooth and clear solution was obtained. Next, rGO-amino-HNT (1.0 g) that had been previously prepared was exfoliated for 30 min by ultrasonication in methanol (10 mL) to form a stable aqueous dispersion. The rGO-amino-HNT dispersion and Co(NO_3_)_2_·6H_2_O and Zn(NO_3_)_2_·6H_2_O solution were added sequentially to 1 g of 2-methylimidazole solution under stirring. The solution was sonicated for 10 min and transferred to a 100 mL stainless steel autoclave, where the autoclave lid was tightly closed and sealed. The autoclave was placed inside an oven at 100 °C for 12 h. Then, the oven temperature was reduced by 4 °C min^−1^ to reach the ambient temperature, when the resulting crystals were separated by centrifugation, washed with methanol and dried at 60 °C. Fig. 1 depicts the different stages of the nanocomposite synthesis.

### 2.7. Preparation of thin film rGO-amino-HNT/Co_0.5_Zn_0.5_(MeIm)_2_

**Fig. 2:**
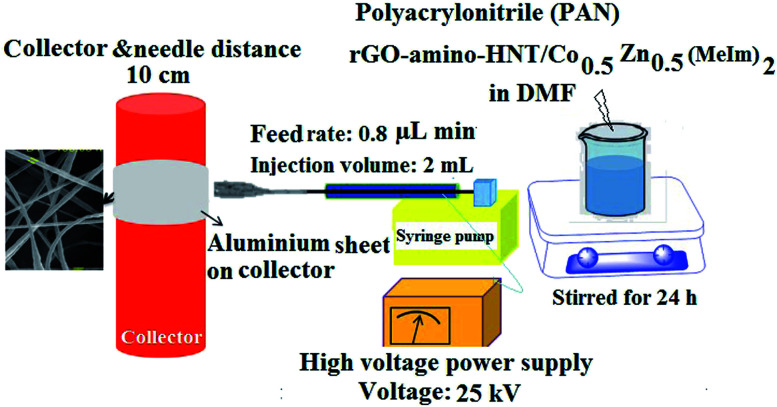
A schematic diagram of the design of the technique used to construct the desired thin film.

To prepare the thin films, 80 mg of rGO-amino-HNT/Co_0.5_ZnO_0.5_(MeIm)_2_ MOF was added to 2.5 mL of DMF and stirred for 4 h to obtain a homogenous solution. After complete dissolution of the polymer and obtaining a homogeneous solution, 1.5 g of polyacrylonitrile was added, and stirred at 600 rpm for 24 h. Then 2 mL of this solution was withdrawn into a 5 mL syringe which was located in a syringe pump. A homemade SPME device was attached to an electrical motor such that the plunger wire could be rotated at 20 cycles per min while electrospinning was performed. Under these conditions, the external needle and the polymer-containing syringe needle were connected to high voltage power supply terminals. Here 1.5 cm of wire from the end part of the plunger was used for collecting the nanofibers. The other part of the SPME assembly was protected from the nanofibers flying toward the collector by a packed paper insulator. The distance between the needle and the collector was set at 10 cm. The SPME assembly was set perpendicular to the syringe. A voltage of 25 kV was applied for producing the nanofibers while a flow rate of 0.8 μL min^−1^ was set for the polymer solution delivery using a syringe pump (Fig. 2).

## 3. Results and discussion

### 3.1. Characterization of thin film rGO-amino-HNT/Co_0.5_Zn_0.5_(MeIm)_2_

**Fig. 3:**
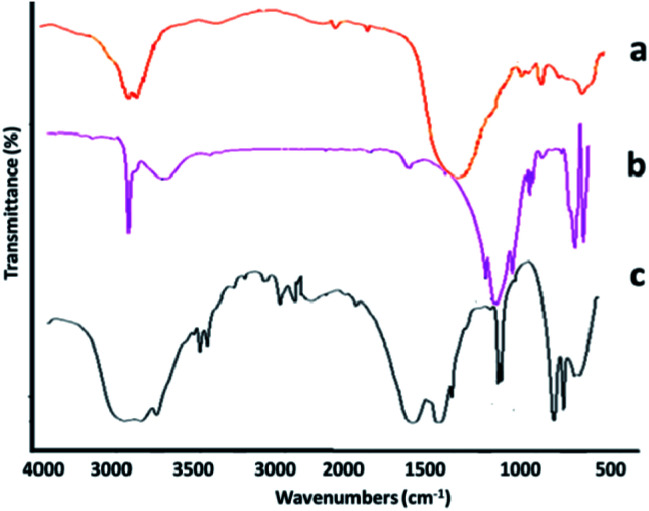
FTIR spectra of (a) halloysite, (b) amino-HNT, and (c) reduced graphene oxide-amino-halloysite/bimetallic metal–organic frameworks.

**Fig. 4:**
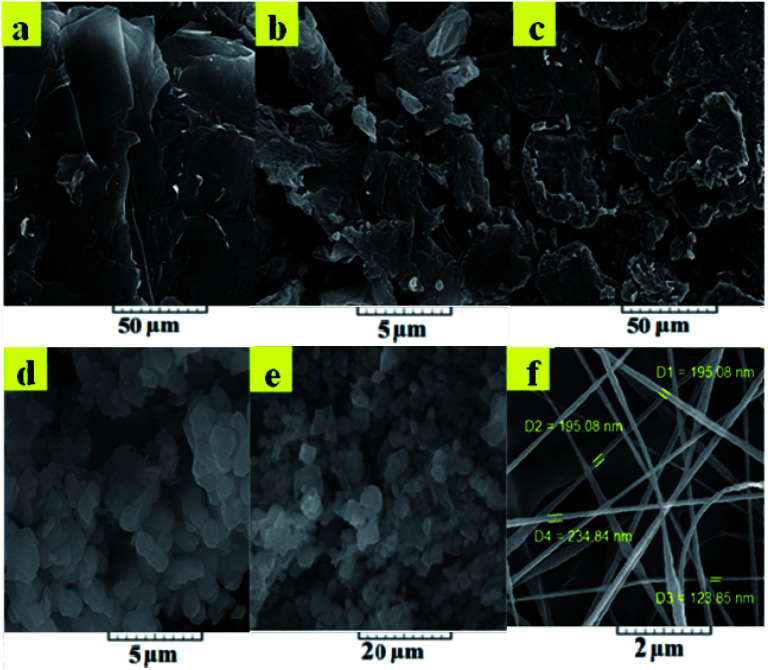
Scanning electron micrographs of (a) graphene oxide, (b) reduced oxide graphene, (c) reduced graphene oxide-amino-halloysite, (d) bimetallic metal–organic framework Co_0.5_Zn_0.5_(MeIm)_2_, (e) reduced graphene oxide-amino-halloysite bimetallic metal–organic frameworks, and (f) nanofiber thin film.

The structural composition of the adsorbent was investigated *via* infrared spectroscopy (Fig. 3a–c). In Fig. 3a, the absorption bands appearing at positions 1885, 1628, 6834, 755, 579, and 418 cm^−1^ are related to the HNT composition. Absorption bands at regions 3642 and 3674 cm^−1^ are assigned to the O–H stretching vibration of the Al–OH surface hydroxyl group, while the absorption bands of 949 cm^−1^ are attributed to decomposition of the upper O–H groups. The peak at 1072 cm^−1^ shows the stretching vibrational plate of the Si–O group. Vertical vibrations Si–O are seen at 754 cm^−1^.^35^ The spectrum in Fig. 3b indicates that the halloysite was successfully functionalized by 3-APTES. This claim is confirmed by the presence of peaks at 3430, 2921, and 2844 cm^−1^. The peaks at 1050, 667, and 1120 cm^−1^ represent the stretching vibration of the C–O group of epoxy and alkoxy. The vibration at 1580 cm^−1^ can be related to the stretching vibration of C=O in rGO. Fig. 3c displays the specific absorption bands in the rGO-amino-HNT/Co_0.5_ZnO_0.5_(MeIm)_2_ MOF. The observed groups consisted of O–C–O bending groups (1567 and 1444 cm^−1^), stretching vibration absorption peaks of group C=O (1689 and 1624 cm^−1^), the presence of bending vibrations inside the C–H group (1028 cm^−1^) and the stretching vibration of the O–H group (3457 cm^−1^), which are related to MeIm, the organic ligand, and also observation peaks in the positions of 709 and 760 cm^−1^, respectively, are related to zinc and cobalt elements of the MOF structure. The absorption peaks observed in the infrared spectrum of the rGO-amino-HNT/Co_0.5_ZnO_0.5_(MeIm)_2_ MOF show a good fit with the infrared spectra of HNT and rGO-amino-HNT. SEM observations of GO, rGO, rGO-amino-HNT, and rGO-amino-HNT/Co_0.5_ZnO_0.5_(MeIm)_2_ MOF, as well as nanofibers prepared by electrospinning are shown in Fig. 4a–f, respectively. Fig. 4a represents the surface of GO sheets without porosity, while Fig. 4b depicts the surface of rGO. As can be seen from Fig. 4b, the surface area and porosity of rGO are higher than those of GO sheets. In Fig. 4c, the distribution of amino-HNT on the surface of rGO is well observed. As can be deduced from Fig. 4c, the nanoparticles of amino-HNT are sandwiched in graphene sheets.

Fig. 4d reveals the hexagonal structure of the bimetallic Co_0.5_ZnO_0.5_(MeIm)_2_ MOF. These results are in good agreement with the earlier reported data.^36^Fig. 4e indicates the growth and distribution of the bimetallic Co_0.5_ZnO_0.5_(MeIm)_2_ MOF on the surface of rGO on the nanometer scale and the average particle size was determined to be within 37–44 nm. Fig. 4f indicates the string structure of the nanofibers. The results clearly show that the rGO-amino-HNT/Co_0.5_ZnO_0.5_(MeIm)_2_ MOF was formed with a uniform structure and hexagonal geometric shape, which was highly porous. The EDX spectrum confirms the results obtained from the SEM. The elemental EDX analysis obtained from HNT and rGO-amino-HNT/Co_0.5_Zn_0.5_(MeIm)_2_ (Fig. S1a and b,†) shows the different percentage compositions of N, C, O, Co, and Zn in Co_0.5_Zn_0.5_(MeIm)_2_, as well as a combination of different percentages of these elements along with Si element in the rGO-amino-HNT/Co_0.5_ZnO_0.5_(MeIm)_2_ MOF. The change in the percentage composition of O and C in the rGO-amino-HNT/Co_0.5_ZnO_0.5_(MeIm)_2_ MOF can be attributed to the presence of GO in the adsorbent. Also, the presence of Si element is due to the presence of HNT in the rGO-amino-HNT/Co_0.5_ZnO_0.5_(MeIm)_2_ MOF.

Next, the structures of rGO, rGO-amino-HNT, bimetallic MOF and rGO-amino-HNT/Co_0.5_ZnO_0.5_(MeIm)_2_ MOF were investigated using an X-ray diffraction technique. The results of the X-ray diffraction analysis are shown in Fig. S2a–c.† As shown in Fig. S2a,† the corresponding peaks at 2*θ* = 12 observed in the reduced graphene oxide spectrum indicate that rGO has a high degree of orientation with the inner distance of the layers being 3.7 Å. In Fig. S2b and c† the X-ray diffraction results from bimetallic MOF and reduced graphene oxide-amino-halloysite/bimetallic metal–organic frameworks are shown. Both compositions have diffraction peaks at 2*θ* = 2.811, 3.301, 5.171, and 8.431. Also, the reflection peaks appearing at 2*θ* = 29 and 2*θ* = 31 are related to Zn and CO, respectively. The results obtained concur with the results presented in previous studies, confirming the successful combination of the various constituents of the thin films.

### 3.2. The ultrasonic-assisted-thin-film microextraction procedure

**Fig. 5:**
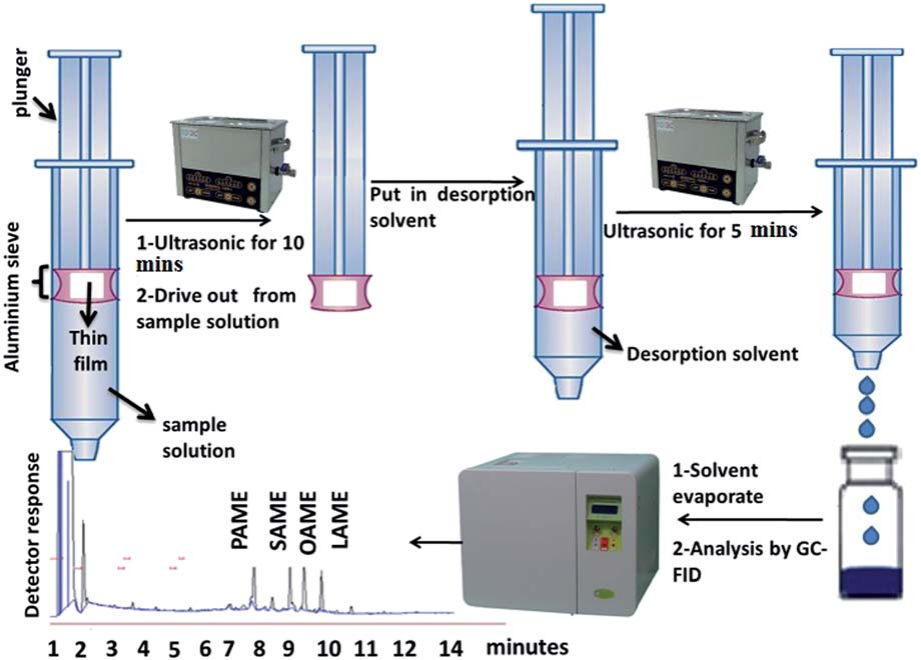
A general picture of the steps of the thin-film microextraction procedure.

The ultrasonic-assisted-thin film microextraction (UA-TFME) was performed as follows: initially, the thin film prepared as described in Section 2.7 was cut into strips 1 cm in width. For each step of extraction, part of the thin film sheet was placed inside a gauze metallic cylinder, and then the set-up was connected to the end piston of the 5 mL syringe. For each stage of extraction, 2 mL of standard solution of fatty acid methyl esters was placed in a 5 mL syringe. Thereafter, the film was attached to the piston immersed in the solution of fatty acid methyl esters, and sonicated for 10 minutes. Then the thin film attached to the piston was removed from the syringe and immersed in another syringe containing 500 μL of desorption solvent (chloroform) and sonicated for 5 minutes. Then, 10 μL of this solution was injected into the gas chromatography system. A general picture of the steps used is shown in Fig. 5.

### 3.3. Optimization of the separation conditions

In the next step, the parameters affecting the extraction efficiency of the UA-TFME method were investigated. Optimization of the parameters was carried out in two stages of desorption and adsorption. First, the desorption parameters, including the properties of the desorption solvent and desorption time were investigated one factor at a time. Then the parameters affecting the adsorption step including pH of solution, extraction time and salt percentage were optimized using a Box–Behnken design and Design-Expert 7 software. To optimize the extraction conditions, a concentration of 10 μg L^−1^ of fatty acid methyl esters was applied and the recovery percentage was considered as the response. The percentage recovery was calculated from the ratio of the peak area of the compounds by using a microextraction process to direct injection of 10 μg L^−1^ of fatty acid methyl esters. All the above experiments were repeated three times.

#### 3.3.1. Optimization of the desorption conditions

In this study, before optimization of the parameters affecting the adsorption stage, parameters controlling the desorption step, such as type and volume of desorption solvent and desorption time, should be optimized. Methods based on desorption are usually carried out in two ways, either desorption by heat or by a solvent. In this work, the desorption process was performed using a solvent. For this purpose, the type and volume of the desorption solvent were evaluated. The desorption solvent has to meet certain requirements: (1) the desorption solvent should have a strong tendency towards the species. In other words, the species should have a desirable and acceptable solubility in the desorption solvent, which requires close polarity of the desorption solvent and species. (2) The desorption solvent should have low volatility and be immiscible in the aqueous phase. (3) The desorption solvent must have good gas chromatographic behavior.

**Fig. 6:**
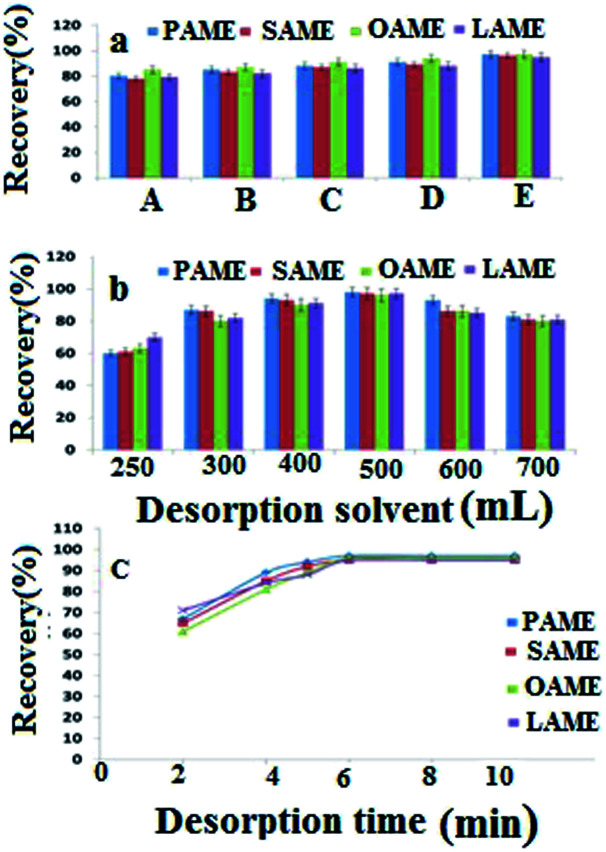
(a) The effects of desorption solvents on the extraction efficiency (A: toluene; B: cyclohexane; C: *n*-hexane; D: dichloromethane; and E: chloroform). (b) The effects of the desorption solvent volume on the extraction efficiency. (c) The effects of the desorption time on the extraction efficiency.

As FAMEs are hydrocarbon compounds with a hydrocarbon chain and are almost non-polar, so the polarization and solubility of the target analytes constitute the most important feature for choosing the desorption solvent. In this study, seven solvents with different solubilities and polarities, hexane, cyclohexane, toluene, dichloromethane, methanol, acetonitrile and chloroform were investigated. The obtained results (Fig. 6a) revealed that chloroform provided higher extraction efficiencies. Thus, chloroform was selected as the appropriate organic solvent for further analysis. Next, the volume of desorption solvent was investigated. The effect of desorption solvent volume on the extraction efficiency of the compounds was within the range of 250 to 700 μL. The results (Fig. 6b) indicated that with an increase in the chloroform volume from 250 to 500 μL, the extraction efficiency of the compounds increased, and then diminished with a further increase in the volume of desorption solvent. As the volume of chloroform increased, the preconcentration factor decreased, whereby the dilution effect would occur. At volumes below 250 μL, the extraction efficiency was reduced since the compounds were not completely absorbed. Hence, 500 μL was selected as the optimal volume for subsequent experiments.

Desorption time is another parameter affecting desorption. In this study, ultrasonication was used for both adsorption and desorption stages. Indeed ultrasonic sound has found increasing applications in the last decade for extraction as well as desorption of compounds. The prominence of the application of ultrasound is a uniform and severe condition for extraction and desorption processes. Because of these remarkable points, the extraction and desorption times normally used for these processes are shorter compared to using a magnetic stirrer or other device.^36,37^ According to preliminary studies, the desorption time was investigated within the range of 2 to 10 min. The results clearly indicate (Fig. 6c) that increasing the time of desorption would boost the extraction efficiency, with the best extraction efficiency of FAME compounds obtained in 6 min. After 6 min, the curve reached a plateau and no increase in the extraction efficiency was observed with additional time. Thus, an extraction time of 6 min was selected for subsequent experiments.

#### 3.3.2. Experimental design and the optimization of the absorption parameters *via* the Box–Behnken technique

In the present study, three factors, pH of solution (pH = 3–11), salt percentage (0–10%) and adsorption time (5–15 minutes), could affect the extraction efficiency of adsorption and were considered as input parameters, while the concentration of 10 μg L^−1^ of FAMEs was maintained as a constant input parameter. Also, the recovery percentage of the target compounds was considered as the experimental response. The pH of the sample solution has a remarkable effect on the extraction efficiency. This is because analytes can be transferred from the aqueous solution to the organic phase in a molecular form. In this case the response is greater than that of analytes in an ionic form. Accordingly, the effect of this parameter was evaluated within a wide range of 3–11. The other main parameter with a major influence on the extraction efficiency is ionic strength. The effect of ionic strength was investigated by adding NaCl in the range of 0–10 (w/v%). Note that adding salt can have a dual effect in extraction techniques with fibers; increasing salt can reduce the extraction efficiency (the salting-in effect) while it can also enhance the extraction efficiency (the salting-out effect). So, salt concentration can be an important factor for extraction efficiency. In addition, extraction time is another important factor that can affect the efficiency of thin film microextraction. The results of preliminary experiments showed that when the extraction time was less than 5 minutes, extraction efficiency was low, while with an extraction time of more than 15 minutes no increase in the extraction efficiency was observed with additional time. Due to the important effects of all the mentioned variables on the extraction efficiency of the method, the Box–Behnken design was used directly for optimizing the conditions without screening. The levels of factors were selected based on initial experiments using one variable at a time. In the case of four to three variables, the Box–Behnken design was selected, as it needs fewer experiments than a central composite design. The Box–Behnken design is suitable for finding quadratic-level responses and for finding a second-order polynomial model which can be expressed as in the following equation:*Y* = *β*_0_ + *β*_1_*A* + *β*_2_*B* + *β*_3_*C* + *β*_12_*AB* + *β*_13_*AC* + *β*_23_*BC* + *β*_11_*A*^2^ + *β*_22_*B*^2^ + *β*_33_*C*^2^*Y* is the estimated coefficient, *β*_0_ is the constant coefficient and *β*_1_, *β*_2_, and *β*_3_ are the linear coefficients which were calculated from the observed values of *Y* from the experiment, and *A*, *B* and *C* are the independent variables. The terms (*AB*, *AC*, *BC*) and (*A*^2^, *B*^2^, *C*^2^) represent the interaction and quadratic factors, respectively. *β*_12_, *β*_13_, *β*_23_ are the coefficients of interaction between the three factors (*A*, *B*, *C*) while *β*_11_, *β*_22_, *β*_33_ indicate second-order coefficients of the parameters. The pH of the solutions (*A*), salt percentage (*B*), and extraction time (*C*) are the independent variables selected to investigate their effect on the extraction efficiency of FAMEs *via* the UA-TFME method. In the adsorption stage, the three independent variables, the pH of the solutions, salt percentage and adsorption time were coded *A*, *B* and *C*. The number of experiments (*N*) could be expressed by the equation *N* = 2*K* (*K* − 1) + *C*_0_, where *K* is the number of variables and *C*_0_ denotes the number of points in the center. In this study, *K* and *C*_0_ were set at 3 and 3 respectively, meaning that 15 experiments had to be done. Table 1 outlines the range and levels of the variables and the response variable. Levels of factors were obtained based on initial tests of one variable at a time. From the 15 experiments, in three experiments, all factors lay within the central levels of each factor. Indeed, these points have been related to the vertices of the cube. After doing the experiments, the average of three replicates from each experiment and the mean of the responses as the final response for each compound were introduced into the software after which the results were analyzed and interpreted. Table 1 reports the range and levels of the variables and the corresponding response variables in the Box–Behnken test. To find the most essential effects and interactions, analysis of variance (ANOVA) was performed using Design-Expert 7 statistical software. Table 2 summarizes the results obtained from the ANOVA study for all target compounds. A *p*-value lower than 0.05 in the ANOVA table indicates statistical significance of the effect at the 95% confidence level. An *F*-test was used to assess the statistical significance of each term in the equation within a confidence interval of 95%. Meanwhile, according to the results in Table 2, the factors of pH of the solutions (*A*) and extraction time (min) (*C*) as well as the interaction of pH–extraction time (*AC*), and square parameters (*A*^2^, *B*^2^ and *C*^2^) had a significant (*p* < 0.05) impact on the microextraction efficiency for the fatty acid methyl esters. In the next step of the design, a response surface model was developed by considering all significant interactions in the BBD. Data analysis offered a semi-empirical expression of *R*% with the following equation:*R* = 95.00 + 0.63*A* − 6.87*B* + 2.00*C* − 1.25*AB* + 1.0*AC* + 5.00*BC* − 6.8*A*^2^ − 3.38*B*^2^ − 1.62*C*^2^

The experimental variables and levels of the Box–Behnken design

**Table:** 

Factor	Level		
	Low (−1)	Central (0)	High (+1)
(A) pH	3	7	11
(B) NaCl (% w/v)	0	5	10
(C) Extraction time (min)	5	10	15

**Table:** 

Run	(*A*)	(*B*)	(*C*)	Recovery (%)
1	0	0	0	96
2	0	+1	+1	86
3	−1	−1	0	90
4	+1	0	−1	85
5	0	0	−1	95
6	+1	−1	0	93
7	−1	0	+1	86
8	+1	+1	0	77
9	0	−1	−1	95
10	0	0	0	94
11	+1	0	+1	90
12	0	−1	+1	99
13	0	0	0	85
14	−1	+1	0	79
15	0	+1	−1	80

**Table:** Analysis of variance (ANOVA) for the BBD

Source	SS	*D*_f_	MS	*F*-Value	*P*-Value	Status
Model	632.08	9	70.23	66.89	<0.0001	Significant
*A* – pH	3.13	1	3.13	2.98	<0.0001	Significant
*B* – NaCl	12.378	1	378.12	360.12	0.1451	Significant
*C* – extraction time	0.32	1	32.0	30.48	0.0027	Significant
*AB*	25.6	1	6.25	5.95	0.0587	Significant
*AC*	4.00	1	4.0	3.81	0.01084	
*BC*	1.00	1	1.00	0.95	0.3799	Significant
*A* ^2^	174.52	1	174.52	166.21	<0.0001	Significant
*B* ^2^	42.06	1	42.06	40.05	0.0015	Significant
*C* ^2^	9.75	1	9.75	9.29	0.0285	Significant
Residual error	5.25	5	1.05			
Lack-of-fit	3.25	3	1.08	1.08	0.5129	Not significant
Pure error	2.00	2	1.00			
Total	637.33	14				
*R*-squared	0.991					
*R*-adjusted	0.976					

On the other hand, based on ANOVA findings in Table 2, the factors of salt concentration (% w/v) (*B*) and also the interactions between factors pH–salt concentration (*AB*) and salt concentration–extraction time (*BC*) had *p* > 0.05 and had non-significant effects on the microextraction efficiency of all fatty acid methyl ester compounds. Thus, these terms were deleted from the second-order quadratic polynomial equation, and the equation is summarized as follows:*R* = 95.00 + 0.63*A* + 2.00*C* + 1.0*AC* − 6.8*A*^2^ − 3.38*B*^2^ − 1.62*C*^2^

According to the obtained model, variables with positive coefficients indicate that with increasing levels of the variable, the response grows. But variables with a negative coefficient indicate that a better response is obtained at lower levels of the variable. According to the obtained equation, pH and extraction time with coefficients of 0.63 and 2.0 had a positive effect on the percentage recovery of the compounds, respectively, while the salt concentration (*B*) showed a negative coefficient and had a diminishing effect on the extraction efficiency. In addition, the coefficients indicated that the most effective interactions were related to the interactions of extraction time and salt concentration and also the second-order terms of all three factors.

The statistical significance corresponding to the quadratic model was predicted by ANOVA based on recovery (%) as the responses (Table 2). The results showed that the *F*-value of the fitted models for the recovery (%) of the four analytes (%) was 66.89. A *p*-value < 0.05 proved the ability and suitability of the model for good and practical prediction of the behavior of all analytes during the microextraction process. The lack of fit test was applied, which determines the failure of the fitted model to represent experimental data in the experimental domain at points not used in the regression equation. The lack of fit *F*-values of the model for the simultaneous microextraction of target analytes was 1.08. The “lack of fit *F*-value” responses showed that the lack of fit values were not significant compared to the pure error. The *p*-value corresponding to the “lack of fit” test for the target analytes was 0.5129; thus, a non-significant “lack of fit” showed that the regression model was strongly significant.

The *R*^2^ of the model of 0.9901 suggests that the sample variation of 99.01% for the simultaneous microextraction of FAME compounds can be ascribed to the independent variables and only 0.03% of the total variation is not described by the model. The adjusted *R*^2^ (0.976) was sufficiently high to indicate the significance of the model. It has been suggested that *R*^2^ should be at least 98% for a good fit of a model. The quadratic model was selected for model development, as suggested by the software (Table 2). A low coefficient of variation (CV%) value is an adequate indication of precision and reliability of the experiments. Indeed, a low CV% of 4.39% suggests good precision and higher reliability of the models for predicting experimental results. The values of the *R*^2^ and adjusted *R*^2^ for the simultaneous microextraction of all fatty acid methyl esters indicated a very good agreement between the experimental data and the predicted values of the responses. It also indicated that the response surface quadratic model was the most adequate for predicting the efficiency of simultaneous microextraction of FAME onto the rGO-amino-HNT/Co_0.5_Zn_0.5_(MeIm)_2_ thin film. This means that the calculated models could explain more than 90% of the results (or of the variability of the response).

The results indicated that the model applied to fit the response variables was significant and appropriate to represent the relationship between the response and the independent variables.

#### 3.3.3. Effects of interactive variables

**Fig. 7:**
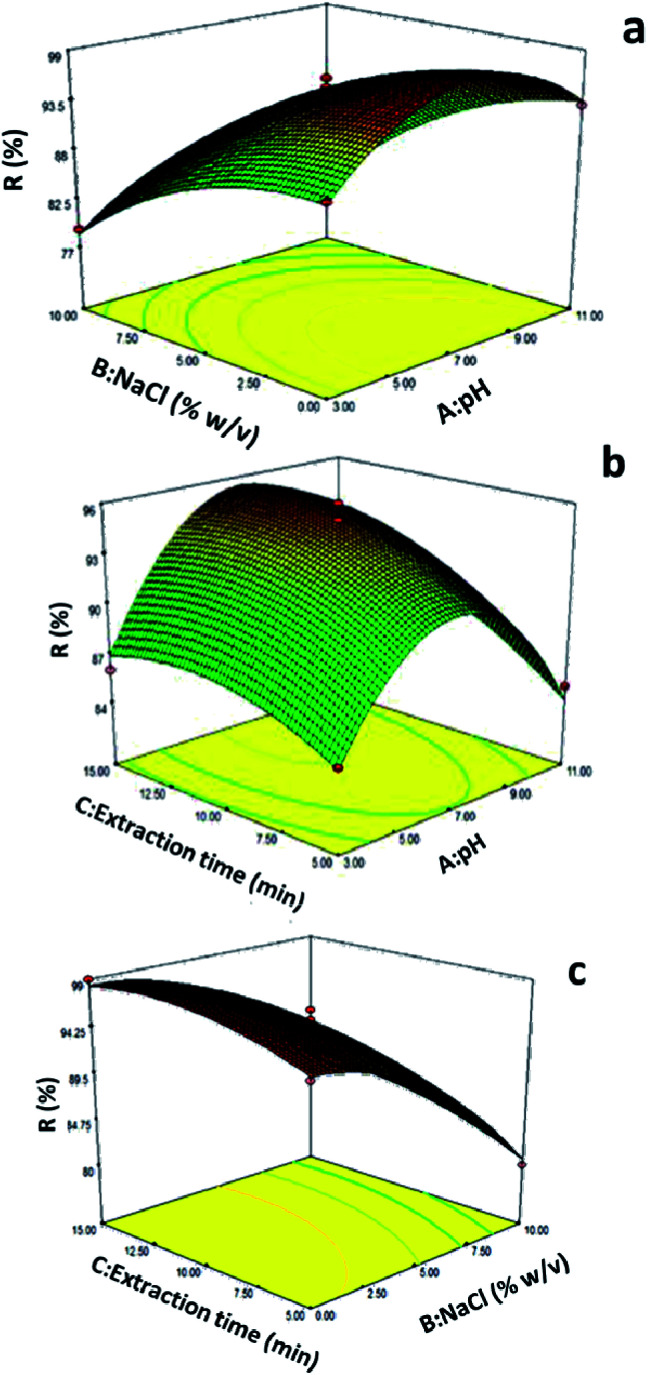
(a) 3D plots of significant factors: (a) pH *vs* extraction time (min); (b) pH *vs.* NaCl (% w/v); and (c) extraction time (min) *vs* NaCl (% w/v).

To explore the effect of independent variables on the dependent variable, three-dimensional response graphs (Fig. 7) were drawn using Design-Expert 7 software and evaluated further. Typically, three-dimensional response charts show the interaction of each of two variables on the response variable for a state where the other variables are constant in the center-point condition. Fig. 7a indicates the interaction between the two parameters of pH and extraction time while the other factors were fixed at their center point. The results indicate a strong interaction between these two factors. The effect of pH on the extraction efficiency of FAME was investigated in the range of 3–11 using phosphate buffer (0.1 mol L^−1^) with the effect of extraction time examined within 5 to 15 min. As can be seen, the highest extraction efficiency was obtained at pH 7 and extraction time within 10 min. The FAMEs have a stereo group that can be hydrolyzed under acidic and alkaline conditions. Hydrolysis destroys the molecular structure of the FAMEs and converts these compounds into other molecules.^38^ According to the results, the recovery percentage and efficiency of extraction of the compounds decreased in acidic and alkaline media. Thus, according to the observed results, the best pH was neutral (pH = 7) to obtain the best yield. Note that the microextraction method is an equilibrium-based technique. Therefore, there is a direct relationship between extracted amounts of analyte and extraction time; hence, the highest amount of extraction efficiency can obtained in the equilibrium state.^39–42^ The influence of extraction time on the extraction efficiency was studied within 5 to 15 minutes. As can be seen in Fig. 7b, in parallel with the increase in the extraction time, the recovery percentage of the corresponding compounds is enhanced, and the maximum responses for all analytes were achieved at an extraction time of 10 min. After 10 min, the curve reached a plateau and no increase in the extraction efficiency was observed with further prolongation.

Fig. 7c displays the interaction between extraction time and ion strength with regards to the extraction efficiency. The results reveal that at an extraction time of 10 minutes and a salt concentration of 0%, the highest extraction efficiency was obtained, while at a time greater than 10 minutes, no significant change was observed in the extraction efficiency, but salt concentrations of more than 0% led to a significant reduction in the extraction efficiency. The effect of sodium chloride on the efficiency of extraction was investigated within the range 0–10% (w/v). It was observed that the best extraction efficiency was obtained when no salt was added to the solution. This effect could be due to the increased viscosity of the soluble mass, which affects the distribution of analyte between the two organic phases and the soluble phase, thus reducing the rate of penetration and distribution of analyte from the soluble mass to the extraction phase.^40^

#### 3.3.4. Optimization of the BBD *via* the desirability function for the preconcentration of analytes

Fig. S3† presents the (DF) profiles of the response surface model. The numerical optimization of the software has been chosen to find the specific point that would maximize the DF. A minimum and a maximum level had to be provided for each parameter admitted in this study, while the curvature of the response surface curves and their combination with desirability functions would sometimes lead to the appearance of some maxima.^41^ The criteria for optimizing all studied factors corresponding with the recoveries are shown in Table 1. A multiple response method was applied for optimizing any combination, including pH (3–11), extraction time (5–15 min) and salt concentration (0–10%) based on the achievement of conditions for reaching the best ideal response (100%). Fig. S3† reveals a desirability ramp which was generated from 10 optimum points *via* numerical optimization. A desirability value of 1.00 indicates that the estimated function can well represent the experimental model and the desired conditions. The best local maximum was found to occur at pH 7, extraction time 10 min and NaCl 0%.

### 3.4. Analytical performance

To evaluate the analytical performance of the PAN/rGO-amino-HNT/Co_0.5_Zn_0.5_(MeIm)_2_ nanofiber film for ultrasonic-assisted thin film microextraction (UA-TFME) of fatty acid methyl esters (FAMEs), the figures of merit of this method, including limit of detection (LOD), linear range, repeatability, reproducibility, and enrichment factor were investigated and the results under the optimum conditions are summarized in Table 3. The calibration plots for all compounds were found to be linear within the range of 0.03–5000 μg L^−1^, with a coefficient of determination (*r*^2^) of 0.997. For each concentration level, three replicate extractions were performed. The sensitivity of the method was assessed by calculating the detection (LOD) (S/N = 3) and quantification (LOQ) (S/N = 10) limits of all fatty acid methyl esters. The LODs for palmitic methyl ester (PAME), oleic methyl ester (OAME), stearic methyl ester (SAME) and linoleic methyl ester (LAME) were within the range 0.03–0.06 μg L^−1^. The LOQ values were calculated for ten replicate runs within the range 0.11–0.23 μg L^−1^. As shown, the proposed method has low LOD and LOQ and can be used for trace analysis in complex samples, such as dairy product milk, yogurt, cheese, yogurt soda and butter samples. In order to determine the accuracy and the extraction recovery of the proposed method, the standard addition tests were performed. The extraction recoveries (*R*) of fatty acid methyl esters were calculated from the following equation:^43^
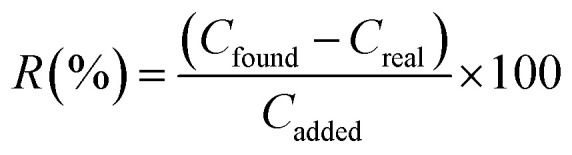
where *C*_found_, *C*_real_, and *C*_added_ represent concentrations of fatty acid methyl ester after adding a known amount of the standard to the real samples, the concentration of fatty acid methyl ester in the real sample and the concentration of a known amount of standard which was spiked into the real sample, respectively.

**Table:** Figures of merit for the proposed method for the determination of fatty acid methyl esters *via* the TFME-GC-FID method

Name	LR (μg L^−1^)	LOD (μg L^−1^)	LOQ (μg L^−1^)	(*R*^2^)	EF^a^	RSD	
						Single film *n* = 5	Film to film
PAME	0.03–500	0.06	0.23	0.997	418	2.1	4.4
SAME	0.03–500	0.06	0.22	0.996	384	2.4	5.3
OAME	0.03–500	0.03	0.11	0.996	387	3.2	3.6
LAME	0.03–500	0.05	0.18	0.997	385	2.5	5.1

a Enrichment factor.

The intra-day and inter-day precisions as well as the accuracies of the assay were evaluated by analyzing samples at three concentration levels on the same day and on five consecutive days. The intra-day and inter-day relative standard deviations (RSDs) were in the range of 2.6–3.4% and 2.4–4.70%, respectively (Table 4). In order to evaluate the reproducibility of the thin film, five replicate determinations were carried out using a single thin film, with the relative standard deviations calculated (Table 4). The results showed that the RSD% values of the method ranged within 3.2–4.4%, indicating that the proposed method is repeatable. Five thin films prepared using identical processes were examined to evaluate fiber reproducibility. The RSD% values (4.6–5.3%) reported in Table 4 indicate the reproducibility of the fibers.

**Table:** Obtained results from the determination of intra-day and inter-day precision values towards fatty acid methyl esters *via* the TFME-GC-FID method

Compound	Added (μg L^−1^)	Found (μg L^−1^)	*R* (%)	RSD (%)	
				Inter-day	Intra-day
PAME	0.15	14.89	99.2	4.7	3.6
	70.0	68.92	98.45	4.4	1.3
	400.0	394.64	98.66	4.2	6.2
SAME	0.15	14.92	98.4	4.7	4.3
	70.0	68.76	98.22	4.4	2.5
	400.0	398.92	99.73	4.2	4.1
OAME	0.15	4.881	99.2	4.7	4.4
	70.0	68.85	98.35	4.4	2.6
	400.0	385.85	96.46	4.2	4.5
LAME	0.15	4.921	99.46	4.7	4.2
	70.0	67.96	97.08	4.2	3.3
	400.0	386.85	96.71	4.7	4.2

The results show that the mean recoveries of analytes, measured at three concentration levels, varied from 95.9 to 100.0% with relative standard deviations of less than 4.7%. The enrichment factors, defined as the ratio of fatty acid methyl ester concentrations after and before extraction, were 418, 384, 387, 385 for palmitic methyl ester (PAME), oleic methyl ester (OAME), stearic methyl ester (SAME) and linoleic methyl ester (LAME), respectively. Fig. 4S† shows the chromatograms of (a) a standard solution of fatty acid methyl esters in *n*-hexane (10 μg L^−1^) and unspiked (b) milk, (c) yogurt, and (d) yogurt soda samples. As can be seen, no significant interference peaks were found at the retention position of these fatty acid methyl esters.

### 3.5. Real sample analysis

The proposed method was applied to the determination of fatty acid methyl esters in different matrices, such as the dairy products, milk, yogurt, cheese, dough, and butter samples. Some fatty acid methyl ester compounds were detected in these samples but some of them were not detected due to the very low concentrations (lower than LOQs). The quantitative results of these samples are listed in Table 5. Fig. 4Sa–d† presents the separation of target analytes in standard solution, and unspiked real samples after extraction using PAN/rGO-amino-HNT/Co_0.5_Zn_0.5_(MeIm)_2_ thin film. The results show that there are no interfering peaks in the chromatograms of the real samples, indicating the ability of the method to be an efficient, clean-up technique. The results of three replicate analyses are listed in Table 5 for real samples spiked with each analyte at three levels 15, 70 and 250 μg L^−1^, respectively. Also, the average concentrations of PAME, SAME, OAME and LAME in milk, yogurt, cheese, yogurt soda, and butter samples are summarized in Table S1.† These results showed that the proposed analytical technique can be applied to real samples with relative recoveries of 95.9–100.0%, suggesting the good accuracy of the method. The RSD values were between 2.4 and 4.7%. These experimental data clearly demonstrate that the novel synthesized PAN/rGO-amino-HNT/Co_0.5_Zn_0.5_(MeIm)_2_ thin film was reliable and suitable for the selective extraction and sensitive determination of trace target analytes in complex samples.

**Table:** Obtained results from the determination of fatty acid methyl esters in spiked dairy product samples *via* the TFME-GC-FID method (*n* = 5)

Sample		Added (μg L^−1^)					
		15		70		250	
		Found (±SD)	*R* (%)	Found	*R* (%)	Found	*R* (%)
Milk	PAME	14.86 (3.3)	99.7 (4.1)	69.92 (3.8)	95.9 (3.2)	249.92 (3.8)	95.5 (3.6)
	SAME	14.73 (4.1)	99.3 (3.6)	64.18 (5.3)	97.6 (3.1)	241.76 (3.3)	97.6 (3.8)
	OAME	14.93 (3.1)	97.8 (4.3)	68.90 (4.1)	95.5 (4.1)	235.97 (4.2)	95.5 (3.6)
	LAME	14.96 (2.7)	99.9 (5.1)	66.89 (4.2)	96.7 (3.2)	241.66 (4.7)	96.7 (3.5)
Yogurt	PAME	14.76 (3.8)	100 (5.4)	66.95 (4.1)	98.4 (4.5)	246.73 (3.3)	98.4 (3.7)
	SAME	14.94 (3.6)	99.8 (4.3)	69.97 (6.6)	95.9 (5.1)	241.97 (5.2)	95.9 (5.6)
	OAME	14.86 (3.4)	100 (3.3)	65.80 (4.8)	98.0 (3.6)	245.23 (4.8)	98.0 (4.3)
	LAME	14.88 (5.1)	97.7 (3.1)	67.52 (6.2)	99.0 (3.8)	245.92 (3.8)	99.0 (4.2)
Cheese	PAME	14.81 (3.3)	97.5 (3.8)	65.96 (5.3)	97.1 (4.7)	245.15 (4.2)	97.1 (3.3)
	SAME	14.87 (2.8)	97.8 (3.4)	69.97 (4.9)	95.9 (5.1)	239.97 (4.2)	95.9 (3.5)
	OAME	14.85 (5.1)	100 (4.4)	65.80 (4.1)	98.0 (6.1)	243.43 (3.8)	98.0 (4.5)
	LAME	14.68 (4.2)	97.7 (3.3)	67.52 (4.7)	99.0 (5.3)	238.68 (5.4)	99.0 (4.6)
Yogurt soda	PAME	14.96 (2.3)	97.5 (5.2)	65.96 (5.3)	97.1 (5.5)	256.14 (5.2)	97.1 (3.7)
	SAME	14.91 (2.8)	99.6 (3.7)	66.15 (6.5)	98.4 (6.3)	244.27 (5.2)	98.4 (4.3)
	OAME	14.87 (3.1)	99.5 (3.5)	67.17 (6.5)	97.1 (5.2)	246.62 (3.8)	97.1 (3.4)
	LAME	14.74 (4.5)	97.2 (3.8)	69.98 (4.7)	95.9 (5.8)	245.55 (3.6)	95.9 (3.2)
Butter	PAME	14.93 (3.4)	97.8 (3.6)	68.90 (6.7)	95.5 (4.8)	235.97 (4.4)	95.5 (5.2)
	SAME	14.81 (4.1)	97.5 (4.7)	65.96 (4.8)	97.1 (4.2)	245.15 (3.8)	97.1 (4.1)
	OAME	14.76 (5.2)	100 (5.6)	66.95 (5.1)	98.4 (3.3)	246.73 (5.2)	98.4 (3.6)
	LAME	14.88 (3.2)	97.7 (3.7)	67.52 (5.5)	99.0 (4.3)	245.92 (2.8)	99.0 (4.5)

### 3.6. Comparison of the proposed method with other related SPME techniques

In this study, for the first time, thin film PAN/rGO-amino-HNT/Co_0.5_Zn_0.5_(MeIm)_2_ was synthesized. Then, the capability of the thin film, in the extraction and measurement of fatty acid methyl esters was investigated by ultrasonic assisted extraction. For this purpose, some statistical data (including LOD, LR, *R*^2^, RSD, and recovery (*R*)) were obtained using fabricated thin nano fiber films to measure the fatty acid methyl ester compounds extracted by the proposed method and then the results were compared with other reported methods (Table 6). According to the results, the proposed method has a wider linear range and lower detection limit than other reported methods. In recent decades, the main aim of researchers in analytical chemistry has been to introduce and develop preparation methods based on the principles of green chemistry. Accordingly, in the present work, the authors introduced a UA-TFME method for the determination of fatty acid methyl esters. As can be seen from the results of Table 6, the proposed method has advantages such as high accuracy and precision, lower detection limit, and wide linear range compared to the previously methods introduced for determining fatty acid methyl esters. Also, other advantages of the proposed method include using a lower sample content, less organic solvent volume, reducing absorption and desorption time, and decreasing the preparation steps; all of the above results correspond perfectly with the approach and objectives of green chemistry.

**Table:** A comparison of the present method studied with other methods for the determination of fatty acid methyl ester compounds

Method	Sample	LR (μg L^−1^)	LOD (μg L^−1^)	RSD	Ref.
Vac-HSSPME-GC-FID^a^	Dairy products	0.005–14 (mg L^−1^)	0.14–1	<10	42
HS-SPME-GC-FID^b^	Cheese	0.3–25	0.15–0.3	<10	29
HS-SPME-GC-MS^c^	Products of *in vitro* fermentation	2.5–50 (mg L^−1^)	1.3–2.1	<5	44
HS-SPME-GC-MS^d^	Zooplanktons	0.5–200	0.01–6.07	<5.7	45
SPME-GC-MS^e^	Lung tissue	0.5–500	0.5–1.1	<4.4	46
HF-LPME-GC-FID^f^	Vegetable oils	10–5000	4.73–13.21 (ng L^−1^)	<12.5	8
In-tube SPME-HPLC-UV^g^	Coffee	0.2–10.0 (mg kg^−1^)	3.0–394.0 (μg kg^−1^)	<14.3	47
HS-SDME-GC-FID^h^	Oxidation products	0.13–850 (mg L^−1^)	0.02–0.3 (mg L^−1^)	<5.0	48
TFME-GC-FID^i^	Dairy products	0.03–5000	0.03–0.06	<5.3	This work

a Vacuum-assisted headspace solid-phase microextraction-gas chromatographic-flame ionization detection.

b Headspace solid-phase microextraction-gas chromatographic-flame ionization detection.

c Headspace solid-phase microextraction-gas chromatographic-mass spectrometry.

d Solid-phase microextraction-gas chromatographic-mass spectrometry.

e Solid-phase microextraction-gas chromatographic-mass spectrometry.

f Hollow fiber liquid-phase microextraction technique, followed by gas chromatography-flame ionization detection (GC-FID).

g In-tube solid-phase microextraction-gas chromatographic-flame ionization detection.

h Headspace single-drop microextraction coupled with gas chromatography flame ionization detection.

i Thin film microextraction-gas chromatography-flame ionization detection.

### 3.7. Quality assurance and quality control with the proposed method

In order to affirm analytical performances of the developed methods (TFME-GC-FID method) under optimized conditions, a review of analytical characteristics of the extraction of FAME from dairy product samples obtained by the proposed method and the method reported by Amer *et al.* (2013).^49^ For this purpose, performance characteristics, including linearity, repeatability, reproducibility, limits of detection (LOD), and quantification (LOQ), were evaluated simultaneously with both methods (the proposed method and Amer *et al.*'s method) by spiking experiments using a FAME-free dough matrix as a representative dairy product. A review of analytical characteristics of the extraction of FAME from dairy product samples obtained by the proposed method and method reported by Amer *et al.*^49^ are listed in Table 7. The calibration curves were investigated with standards addition methods in the levels from 0.03 to 500.0 μg L^−1^. The calibration curves were constructed by using the peak area of analytes at ten different concentrations. The correlation coefficients (*R*^2^) for the analytes were greater than 0.996. LODs and LOQs were calculated as the minimum concentration of an analyte giving peaks in which signal-to-noise ratios are 3 and 10, respectively of individual peaks. LOD and LOQ were in the range of 166.0–250.0 μg L^−1^ and 400.0–500.0 μg L^−1^, respectively. The results suggested that the performance of the developed method (UA-TFME-GC-FID) provides an effective and reliable solution for thin film microextraction and detection of the low concentration of target analytes in different complex matrices. The proposed method gives comparable analytical results to previously reported technique and can afford high selectivity and sensitivity, good extraction efficiency, a wide linear range, and better precision relative to the results obtained by Amer *et al.*'s method. Indeed, the advantages of this method clearly surpass those of reference method. In addition, the method based on UA-TFME-GC-FID has the advantages over previously reported methods of less solvent consumption, low operation time, reduced procedure, and no requirement to add further purification procedures for removing interference.

**Table:** Quality assurance of the proposed method, comparing with the method reported by Amer *et al.* (2013)

Compound	Validation according to the Amer *et al.* (2013) method^49^			This work		
	Linear range (μg L^−1^)	*R* ^2^	LOD (μg L^−1^)	Linear range (μg L^−1^)	*R* ^2^	LOD (μg L^−1^)
PAME	500.0–50 000	0.992	166.0	0.03–500	0.997	0.06
SAME	500.0–50 000	0.995	200.0	0.03–500	0.996	0.06
OAME	500.0–50 000	0.993	250.0	0.03–500	0.996	0.03
LAME	500.0–50 000	0.995	250.0	0.03–500	0.997	0.05

## 4. Conclusions

In this study, novel PAN/rGO-amino-HNT/Co_0.5_Zn_0.5_(MeIm)_2_ nanofiber film was prepared *via* an electrospinning technique. The electrospun nanofibers presented a porous fibrous structure, large surface area, good stability, strong hydrophobicity, and excellent extraction efficiency (EF: 484–418). With PAN/rGO-amino-HNT/Co_0.5_Zn_0.5_(MeIm)_2_ nanofibers as an adsorbent, a UA-TFME-GC-FID method was established and applied successfully for determining fatty acid methyl esters in dairy product (milk, yogurt, cheese, yogurt soda, and butter) samples. The fabricated nanofiber film was stable, inexpensive, and easily prepared, and it could be used more than 30 times without a decrease in extraction recoveries. The proposed method enjoyed advantages such as rapidity, simplicity, and low-cost sample preparation, with acceptable efficiency (*R*: 95.9 to 100.0%) and a low matrix effect compared with other techniques reported for determining fatty acid methyl esters. In addition, the consumption of toxic organic solvents (500 μL) was minimized without affecting the method sensitivity. One of the prominent points of this method is the use of PAN/rGO-amino-HNT/Co_0.5_Zn_0.5_(MeIm)_2_ as an adsorbent employed in UA-TFME for fatty acid methyl esters. Another remarkable feature of the proposed method is its high sensitivity and capacity, due to the use of the highly porous material of the metal–organic framework nanofibers, and the use of ultrasound in both the adsorption and desorption stages that leads to a shorter processing time than magnetic stirrers or other devices. Finally, the proposed method has a very low detection limit (LOD: 0.03–0.06 μg L^−1^) and a better linear range (LR: 0.03–500 μg L^−1^) than other methods reported for FAME determination.

## Conflicts of interest

There are no conflicts to declare.

## Supplementary Material

RA-011-D0RA07674K-s001
