# Supplementary material for: The fabrication of a novel polyacrylonitrile/reduced graphene oxide-amino-halloysite/bimetallic metal–organic framework electrospun nanofiber adsorbent for the ultrasonic-assisted thin-film microextraction of fatty acid methyl esters in dairy products with gas chromatography-flame ionization detection
Source: RSC Adv. 2021 Apr 21;11(24):14686–99. doi: 10.1039/d0ra07674k (PMC8697828; doi:10.1039/d0ra07674k)
Supplement: RA-011-D0RA07674K-s001 [file RA-011-D0RA07674K-s001.pdf]

Electronic Supplementary Material (ESI) for RSC Advances.
This journal is © The Royal Society of Chemistry 2021

Supplementary Materials

The fabrication of a novel polyacrylonitrile/reduced graphene oxide-amino-halloysite/bimetallic metal–organic framework electrospun nanofiber adsorbent for the ultrasonic-assisted thin-film microextraction of fatty acid methyl esters in dairy products with gas chromatography-flame ionization detection

R. Mirzajani[a]*, F. Kardani[a], Z. Ramezani [b]

[a]Chemistry Department, College of Science, Shahid Chamran University of Ahvaz, Ahvaz, Iran

[b] Faculty of Pharmacy, Ahvaz Jundishapur University of Medical Sciences, Ahvaz, Iran

E-mail address: rmirzajani@scu.ac.ir (R. Mirzajani) Tel. / Fax +986133738044

Figurs of contents in Supporting Information:

Fig 1S. EDS spectera of (a) HNT (b) rGO-amino-HNT/ ($Co_{0.5}$ $Zn_{0.5}$ $(MeIm)_2$) nanocomposite.
Fig 2S. X-ray diffraction patterns of (a) reduced graphene oxide (b) bimetallic ZIFs ($Cox{\cdot}Zn_{1-x}(MeIm)_2$) metal-organic framework (c) rGO-amino-HNT/ ($Co_{0.5}$ $Zn_{0.5}$ $(MeIm)_2$) nanocomposite.
Fig 3S. Desirability ramp for the numerical optimization of three goals, namely the pH, NaCl (%w/v), and Extraction time (min).

Fig. 4S Chromatograms of (a) standard solution of fatty acids methyl esters in n-hexane and unspiked, (b) milk, (c) yogurt and (d) yogurt soda.

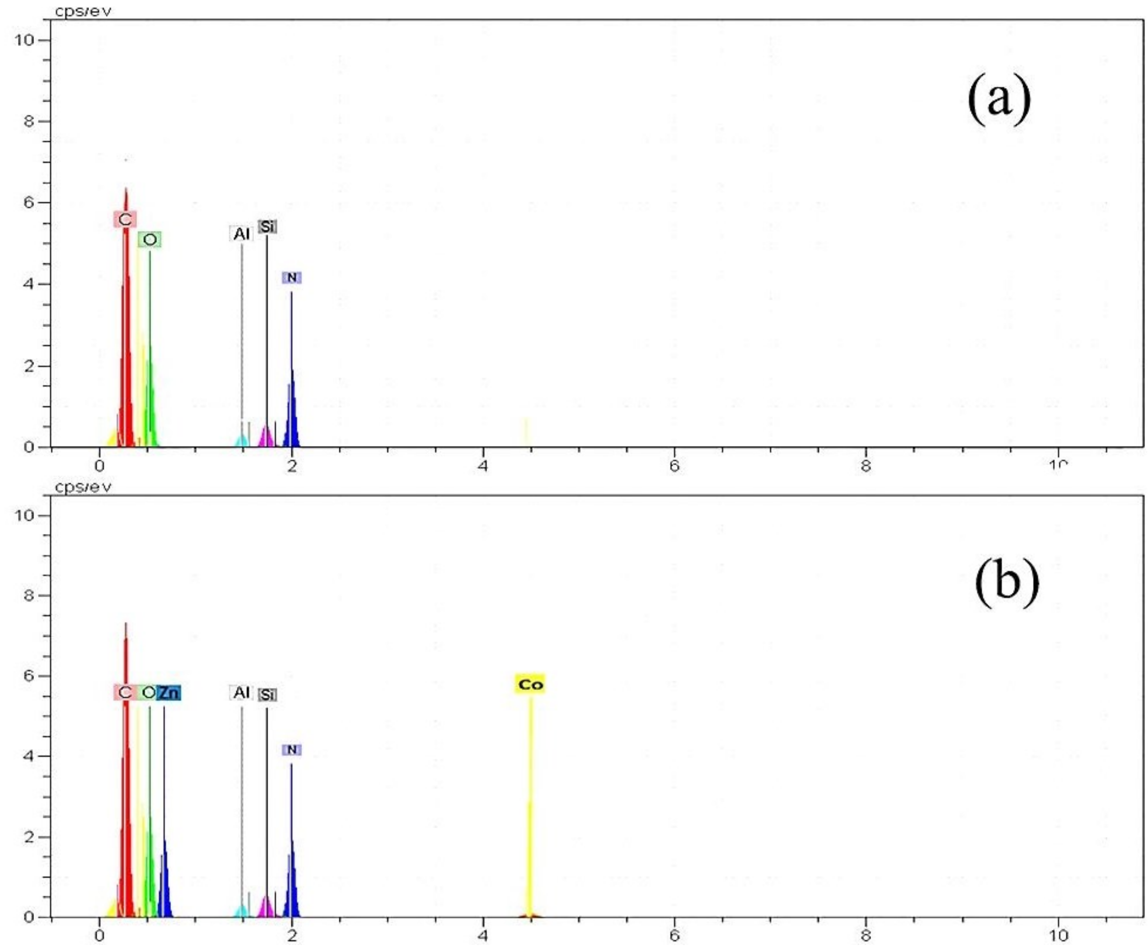

Fig. 1S EDS spectera of (a), HNT (b) rGO-amino-HNT/ ($Co_{0.5}$ $Zn_{0.5}$ $(MeIm)_2$) nanocomposite

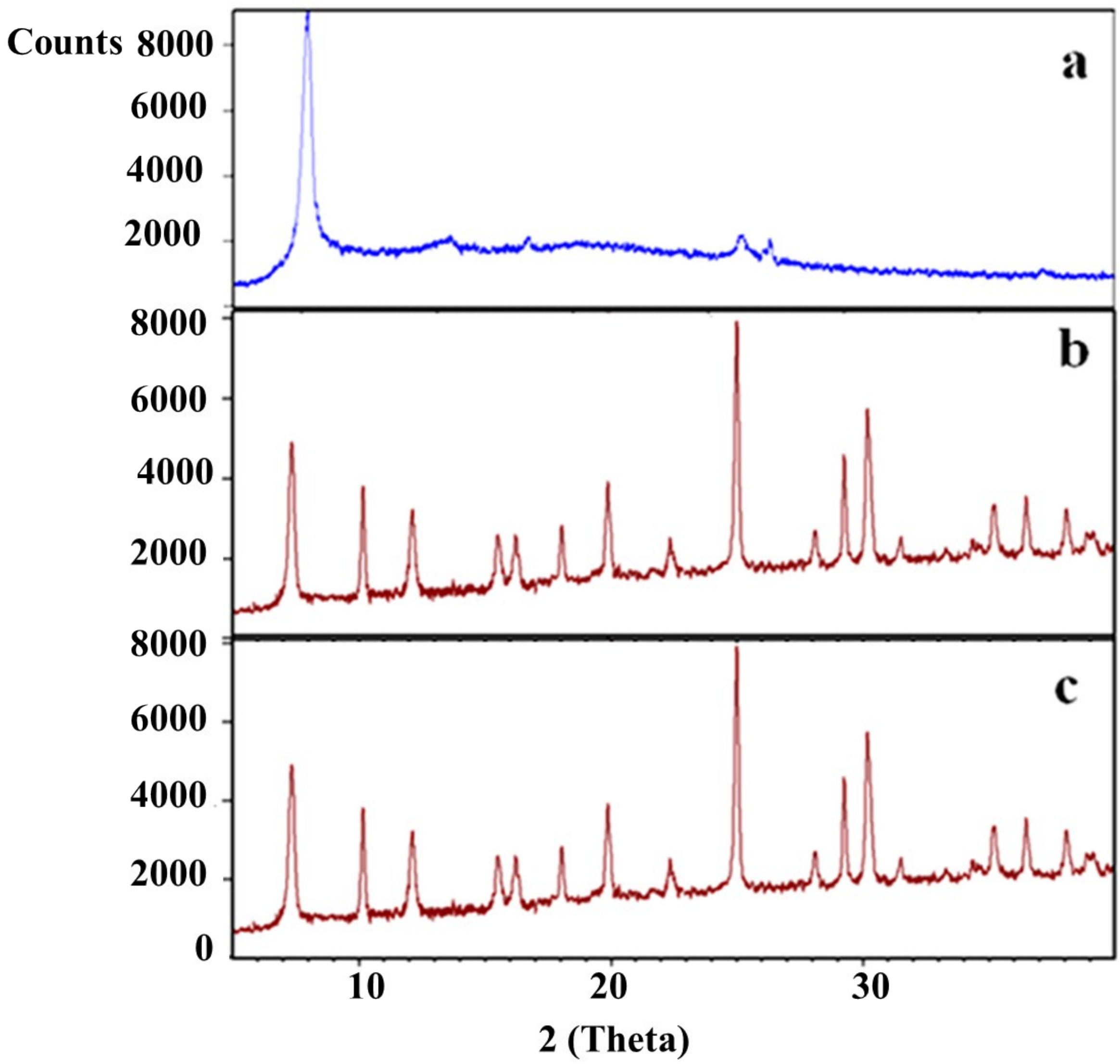

Fig .2S X-ray diffraction patterns of (a) reduced graphene oxide (b) bimetallic ZIFs ($Cox{\cdot}Zn_{1-x}(MeIm)_2$) metal-organic framework (c) rGO-amino-HNT/ ($Co_{0.5}$ $Zn_{0.5}$ $(MeIm)_2$) nanocomposite

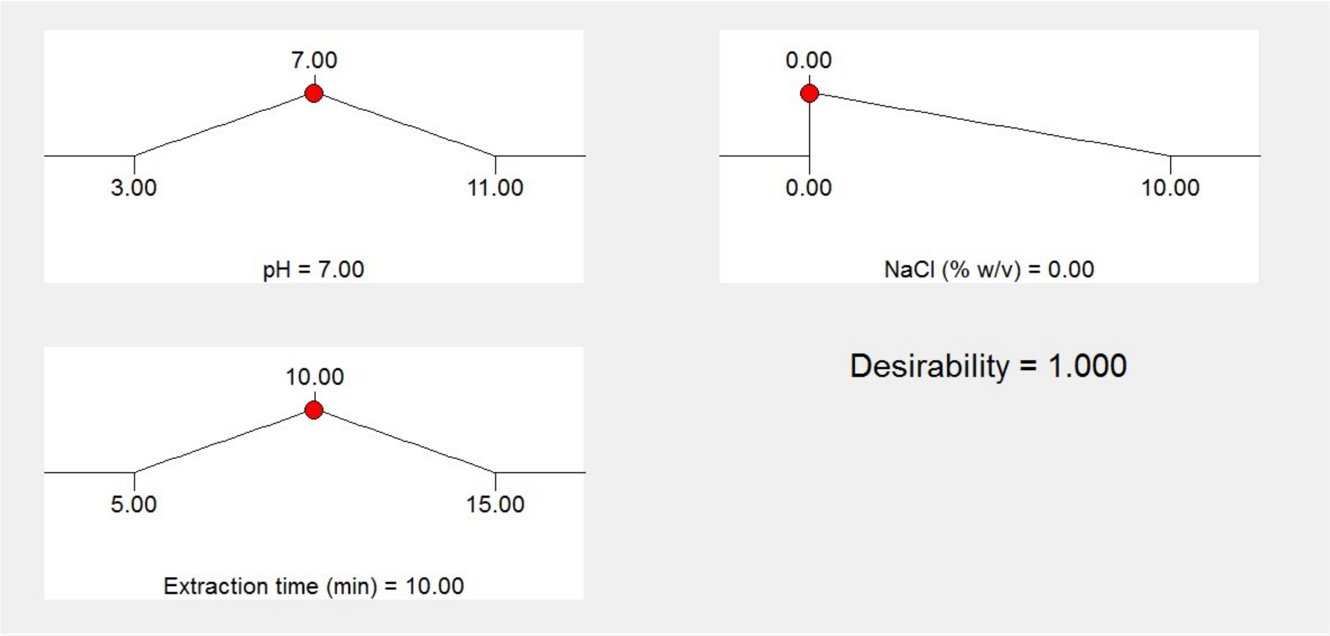

Fig. 3S Desirability ramp for the numerical optimization of three goals, namely the pH, NaCl (%w/v) and Extraction time (min)

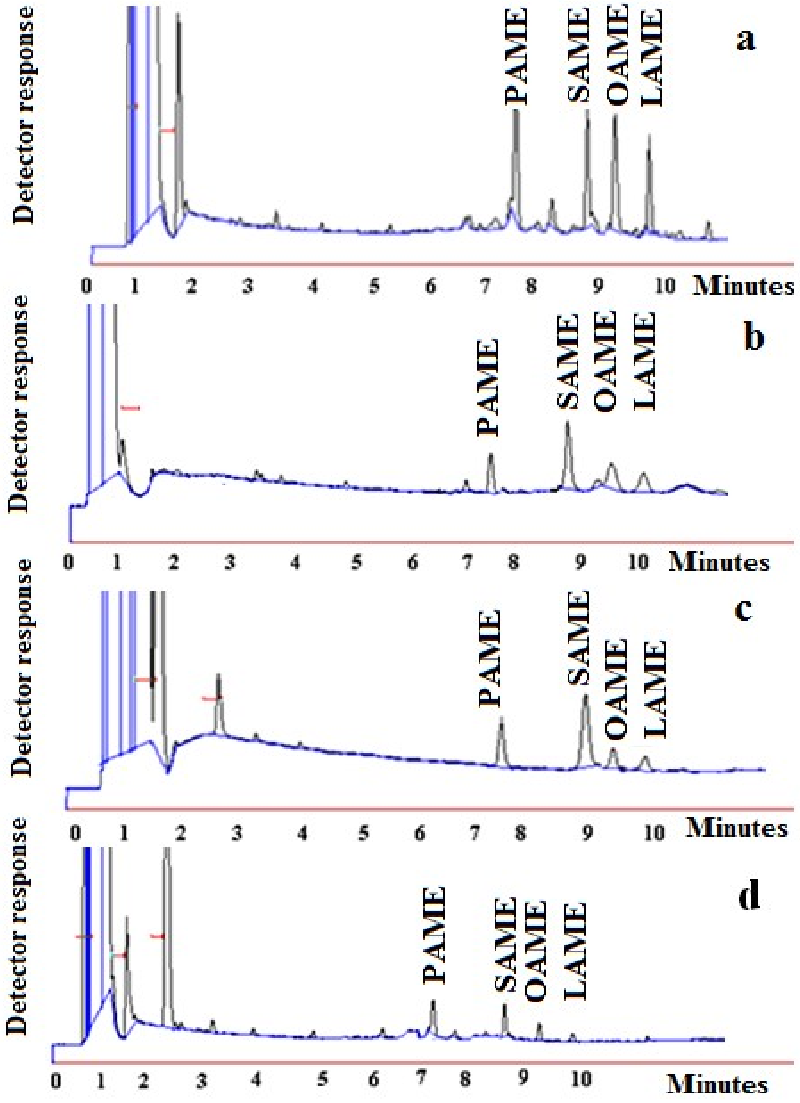

Fig. 4S Chromatograms of (a) standard solution of fatty acids methyl esters in n-hexane and unspiked: (b) milk, (c) yogurt and (d) yogurt soda.

Table S1 The amount of detected of FAME in dairy products using the proposed TFME-GC-FID method

	Concentration (mg kg^{-1})			
**Sample**	**PAME**	**SAME**	**OAME**	**LAME**
Milk	47.35	55.76	28.48	**19.52**
Yogurt	42.62	51.48	18.36	**9.56**
Cheese	38.63	43.58	16.85	**11.85**
Yogurt soda	17.59	22.61	8.47	**7.75**
Butter	63.58	68.43	25.85	**22.57**
